# Thermal Conductivities of Crosslinked Polyisoprene and Polybutadiene from Molecular Dynamics Simulations

**DOI:** 10.3390/polym13030315

**Published:** 2021-01-20

**Authors:** Aleksandr Vasilev, Tommy Lorenz, Cornelia Breitkopf

**Affiliations:** Chair of Technical Thermodynamics, Technische Universität Dresden, 01069 Dresden, Germany; tommy.lorenz@tu-dresden.de (T.L.); cornelia.breitkopf@tu-dresden.de (C.B.)

**Keywords:** molecular dynamics simulations, force field, rubber, polyisoprene, polybutadiene, thermal conductivity

## Abstract

For the first time, the thermal conductivities of vulcanized polybutadiene and polyisoprene have been investigated according to their degree of crosslinking. The C-C and C-S-S-C crosslink bridges, which can be obtained via vulcanization processes using peroxides and sulfur, respectively, are considered. The temperature dependence of the thermal conductivity of soft rubber derived from molecular dynamics (MD) simulations is in very good agreement with the experimental results. The contributions of bonded and non-bonded interactions in the MD simulations and their influence on the thermal conductivities of polyisoprene and polybutadiene are presented. The details are discussed in this paper.

## 1. Introduction

Rubbers are widely used in industry due to their scalable mechanical properties and low masses. Thus, recently, polymers have also served as a basis for interactive fiber–rubber composites consisting of reinforced fibers, shape memory alloys, sensor networks, and magnetic fillers. To predict their thermo-mechanical behavior, experiments and simulations need to be combined. To solve conservation equations with the finite element method (FEM), the thermal properties of each component of the composite as a function of temperature and pressure must be known. These functions can be obtained either from experiments or from theoretical methods, such as molecular dynamics simulations.

Molecular dynamics simulations are a popular tool for investigating the thermal conductivities of polymers [1,2,3,4,5,6,7,8,9]. A linear dependence of the thermal conductivity on the number of degrees of freedom per repeat unit has been found for polyamide-6,6 [7]. An enhancement of the thermal conductivity of polymers through an increase in molecular weight has been found [2,3], which is in agreement with other theoretical [10,11] and experimental [12,13] data. It has been noticed that, at polymerization degrees above 140, heat transfer is dominated by phonon transport [3]. The influence of the degree of crosslinking on the thermal conductivity of polymers has been investigated [14,15,16,17]. For all simulated polymers, the thermal conductivity increases with an increase in the degree of crosslinking. This agrees well with the theory obtained from the network model, where heat conduction through van der Waals and primary bonds is considered [18].

On the other hand, for some polymers, such as polystyrene in Ref. [15] and epoxy resin in Ref. [19], crosslinking does not significantly increase the thermal conductivity. In Ref. [20], it was found that only short crosslink bridges enhance the thermal conductivity. In this case, the crosslinked chains get close to each other; therefore, non-bonded interactions between these chains transfer more heat. It was revealed that with an increase in the degree of crosslinking, the contribution of the non-bonded interactions to the thermal conductivity becomes much higher than the contribution of covalent bonds. This phenomenon has been observed in epoxy resins, where it was revealed that non-bonded interactions are dominant in heat transport [19]. If the distance between the crosslinked chains is higher than 2.5 σ (parameter of the Lennard–Jones potential), then the van der Waals forces are negligible [21].

There are only few research examples in which MD simulations have been used to calculate the thermal conductivities of polybutadiene and polyisoprene, which are widely used as polymer matrixes for rubbers. The thermal conductivities of crosslinked natural rubber with different phr (parts per hundred rubber) of S have been found as functions of temperature [6]. The united atom force field from Ref. [22] was used in this research. The results were compared with experimental data of soft rubber containing 2.5 phr of sulfur and hard rubber containing 47 phr of sulfur [23]. However, these calculated thermal conductivities were roughly two times less than the experimental values. In Ref. [4], the thermal conductivity of untreated natural rubber was calculated. In this research, an adaptive inter-molecular reactive empirical bond order (AIREBO [24]) potential was used to describe the interactions between the atoms, so the hydrogen atoms were modeled explicitly. The results were two times larger than the experimental value.

In Ref. [25], the thermal conductivities of untreated polyisoprene and polybutadiene were found as functions of temperature from MD simulations. The OPLS-UA (United Atom) [26] force field was used to describe the interactions between the atoms. Dihedral and some angle interactions were not considered because the parameters of the united atom force field do not exist for these polymers. It can be concluded that there is a great need for developing accurate force fields for MD simulations of polymers and for more investigations of the nature of heat transport in crosslinked polymers.

Therefore, new modified force fields based on the force fields used in Ref. [25] were developed for MD simulations to find the dependence of the thermal conductivity on the degree of crosslinking for vulcanized polyisoprene and polybutadiene. The results are important for further simulations of elastomers on the macro scale with the finite element method (FEM), in which polyisoprene and polybutadiene are used as polymer matrixes. The findings of this research are also interesting for experimentalists in choosing the type of vulcanization for synthesizing rubbers based on polyisoprene and polybutadiene.

## 2. Simulation Details

**Figure 1:**
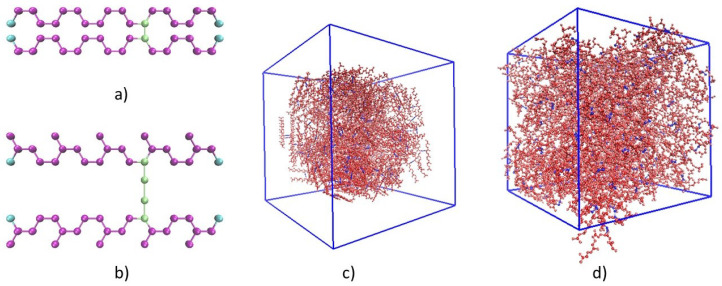
(**a**) Input structure of a molecule for the MD simulation of polybutadiene crosslinked by C-C bridges with a degree of crosslinking of 20%; (**b**) input structure of a molecule for MD simulation of polyisoprene crosslinked by C-S-S-C bridges with a degree of crosslinking of 20%; (**c**) input molecules distributed randomly in the periodic supercell for modeling of polyisoprene vulcanized by sulfur with a degree of crosslinking of 20%; (**d**) structure of polyisoprene vulcanized by sulfur with a degree of crosslinking of 20% before calculation of the thermal conductivity.

The Moltemplate [27] software was used to create cis-1,4-polyisoprene and cis-1,4-polybutadiene chains consisting of 200 monomer units for modeling untreated polyisoprene and polybutadiene. For the simulation of vulcanized polyisoprene and polybutadiene molecules, crosslink bridges were prepared with the Moltemplate software as well. Two types of crosslinking bridges were considered: sulfur bridges (C-S-S-C) as in Ref. [6] and C-C bridges as in Refs. [28,29,30]. Molecules with degrees of crosslinking of 10%, 20%, 33%, 50%, 67%, and 80% were prepared. In Figure 1a,b, the molecules for modeling vulcanized polybutadiene (C-C bridges) and polyisoprene (C-S-S-C bridges) with a degree of crosslinking of 20% are shown. The degree of crosslinking (DC) was defined, as in Ref. [15], by equation

(1) $$\mathrm{DC}=\frac{2N_{\mathrm{CL}}}{N_{\mathrm{mono}}}\cdot 100\%,$$

where $N_{\mathrm{CL}}$ and $N_{\mathrm{mono}}$ denote the total numbers of crosslink bridges and monomer units, respectively. The molecules were randomly distributed in a periodic supercell with the Packmol [31] software (see, as an example, Figure 1c).

A united-atom force field was applied to model the polymeric systems. CH, CH${}_{2}$, and CH${}_{3}$ groups were simulated as “one atom”. The total potential energy of a polymeric system is calculated in this approximation as

(2) $$E=E_{\mathrm{bond}}+E_{\mathrm{angle}}+E_{\mathrm{dihedral}}+E_{\mathrm{non}\text{-}\mathrm{bonded}}.$$

Non-bonded interactions were modeled only with van der Waals interactions. The cutoff distance was set to 10 Å in all simulations. The Lorentz–Berthelot mixing rules were used to find the missing parameters of the Lennard–Jones potential. All simulations were carried out using the LAMMPS [32] software package.

Schematic illustrations of the crosslink bridges and force field parameters used for the MD simulations of polyisoprene and polybutadiene vulcanized by C-S-S-C and C-C bridges are presented in Figure S1 and Tables S1–S4 in the Supplementary Material. Bond stretching and van der Waals interactions were modeled with parameters of the OPLS-UA (United Atom) [26] force field taken from the Moltemplate software. These parameters were used to calculate the thermal conductivities of untreated polyisoprene and polybutadiene [25]. In Ref. [25], however, dihedral and some angle interactions were not considered because parameters of the united atom force field do not exist for these polymers. In the modified force fields used for this research, the parameters for dihedral and angle interactions from the OPLS-AA (All Atom) [26] force field were used to eliminate this problem. The coefficients for special bonds were set to zero in all simulations.

After distribution of the molecules in the periodic supercell, they were polymerized in an NVT ensemble via an algorithm similar to that of Ref. [33]. Each molecule had four CH${}_{3}$ groups in the edges. During this procedure, CH${}_{3}$ groups participating in the creation of a bond between the molecules were turned into CH${}_{2}$ groups. In addition, new bonds, angles, and dihedrals were taken into account for the next time steps of the simulation after creating the new bonds between the molecules. If *M* is the total number of molecules in the periodic supercell and *N* is the number of new bonds created between the molecules during the polymerization algorithm, then *x* = 2N/*M* is the average number of neighbor molecules connected to one molecule. The maximal possible value of *x* is four. On average, for all simulated systems of vulcanized polyisoprene and polybutadiene, *x* was equal to 2.22.

Then, the polymeric systems were heated to a high temperature and were slowly cooled by applying high pressure until normal conditions were reached in an NPT ensemble with a time step of 0.2 fs. The procedure of cooling under pressure was performed three times. Nose and Hoover’s [34,35] thermostat and barostat with damping parameters of 100 and 1000 time steps, respectively, were used to reach the desired temperature, pressure, and density of the simulated systems.

Before calculating the thermal conductivity, the polymeric systems were simulated for 100 ps in an NPT ensemble at the desired temperature and atmospheric pressure with a time step of 1 fs to get the equilibrated density and temperature. After that, the systems were modeled in an NVE ensemble for 900 ps with a time step of 1 fs. During this procedure, at each time step, the total heat flux was calculated. By using the Green–Kubo formula, the thermal conductivity of an isotropic material can be found as

(3) $$\lambda =\frac{V}{3k_{B}T^{2}}\int_{0}^{\infty }\;<\vec{J}\left(0\right)\vec{J}\left(t\right)>dt,$$

where $\vec{J}$ is the heat flux calculated with the following equation taken from Ref. [36]:

(4) $$\vec{J}=\frac{1}{V}\left[\sum_{i}e_{i}\vec{\upsilon }-\sum_{i}S_{i}\vec{\upsilon }\right],$$

where $e_{i}$ is the total energy of the *i*-th atom. The first term is the convectional part of the total heat flux, which represents the heat flux due to the movement of atoms in the system. $S_{i}$ is the per-atom stress tensor calculated with the equation [37]:

(5) $$S_{ab}=-m\upsilon_{a}\upsilon_{b}-W_{ab},$$

where *a* and *b* take on the values *x*, *y*, and *z*, and $W_{ab}$ is the virial contribution, calculated as [37]:

(6) $$W_{ab}=\sum_{n=1}^{N_{p}}r_{I0_{a}}F_{I_{b}}+\sum_{n=1}^{N_{b}}r_{I0_{a}}F_{I_{b}}+\sum_{n=1}^{N_{a}}r_{I0_{a}}F_{I_{b}}+\sum_{n=1}^{N_{d}}r_{I0_{a}}F_{I_{b}}+\sum_{n=1}^{N_{i}}r_{I0_{a}}F_{I_{b}},$$

where $N_{p}$ is the number of neighbors of atom *I* that act on atom *I* via van der Waals interaction, and $N_{b}$, $N_{a}$, $N_{d}$, and $N_{i}$ are the numbers of bonds, angles, dihedrals, and impropers, respectively, and the atom *I* is included in these interactions. $F_{I}$ is the force acting on atom *I* due to these interactions, and $r_{I0}$ is the relative position of the atom *I* with respect to the geometric center of the interacting atoms.

Due to the discretization of time in MD simulations, Equation (4) can be written as follows [38]:

(7) $$\lambda \left(\tau_{M}\right)=\frac{V\Delta t}{3k_{B}T^{2}}\sum_{m=1}^{M}\frac{1}{\left(N-m\right)}\sum_{n=1}^{N-m}J_{i}\left(n\right)J_{j}\left(m+n\right),$$

where $\lambda (\tau_{M})$ is the thermal conductivity obtained from summation to time step *M* (*M* = 0, 1, …, *N*−1), *N* is the total number of simulation steps, and $\tau_{M}$ = *M*Δt.

Models of untreated polyisoprene and polybutadiene with 12,000, 24,000, and 48,000 atoms were prepared and tested to find an optimal size of the periodic supercell for MD simulations of vulcanized polyisoprene and polybutadiene. In the case of untreated polyisoprene, the thermal conductivities for the systems with 12,000, 24,000, and 48,000 atoms were 0.15, 0.149, and 0.154 W/m/K, respectively. For untreated polybutadiene, the thermal conductivities of the systems with 12,000, 24,000, and 48,000 atoms were 0.209, 0.204, and 0.213 W/m/K, respectively. Consequently, the systems with 12,000 atoms were used for MD simulations of vulcanized polyisoprene and polybutadiene.

## 3. Results and Discussion

**Figure 2:**
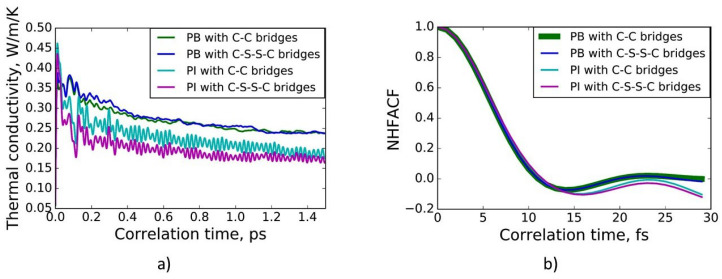
(**a**) Thermal conductivities of polybutadiene crosslinked by C-C bridges (green line) and by C-S-S-C bridges (blue line) and polyisoprene vulcanized by C-C bridges (cyan line) and by C-S-S-C bridges (magenta line) at normal conditions as a function of correlation time; (**b**) the first minima of the normalized heat flux autocorrelation functions (NHFACFs) of polybutadiene crosslinked by C-C bridges (green line) and by C-S-S-C bridges (blue line) and polyisoprene vulcanized by C-C bridges (cyan line) and by C-S-S-C bridges (magenta line) at normal conditions.

The thermal conductivities of crosslinked polybutadiene and polyisoprene with a degree of crosslinking of 20% are presented in Figure 2a. The data for the analysis were taken from the last correlation time interval. As shown in Figure 2a, a correlation length of 1.5 ps was sufficient for convergence of the thermal conductivities. The thermal conductivities along each direction were compared. It was observed that they were equal in all directions. Thus, the models of the crosslinked polymers in the MD simulations were isotropic.

Using the one-dimensional harmonic oscillator model [6], the mechanism of heat transfer was derived. The first minima of the normalized heat flux autocorrelation functions of the vulcanized polyisoprene and polybutadiene with a degree of crosslinking of 20% are presented in Figure 2b. For the crosslinked polyisoprene, the first minimum (see Figure 2b) of the normalized heat flux autocorrelation function is located at *t* ≈ 16 fs, which corresponds to a wavenumber of $\bar{\nu }$ ≈ 332 cm${}^{-1}$. This is close to the wavenumber of C-C-C deformation vibrations in cis-1,4-polyisoprene ($\bar{\nu }$ = 390 cm${}^{-1}$) [39]. For vulcanized polybutadiene, the first minimum (see Figure 2b) of the normalized heat flux autocorrelation function is located at *t* ≈ 14 fs and matches a wavenumber of $\bar{\nu }$ ≈ 379 cm${}^{-1}$, which is close to the wavenumbers of the C-C-C deformation vibrations of cis-1,4-polybutadiene ($\bar{\nu }$ ≈ 405 cm${}^{-1}$) and trans-1,4-polybutadiene ($\bar{\nu }$ ≈ 439 cm${}^{-1}$) [40]. As a result, the heat in crosslinked polyisoprene and polybutadiene is mainly transferred by low-frequency phonons, which correspond to low-energy C-C-C deformation vibrations. This is in agreement with Ref. [3].

**Figure 3:**
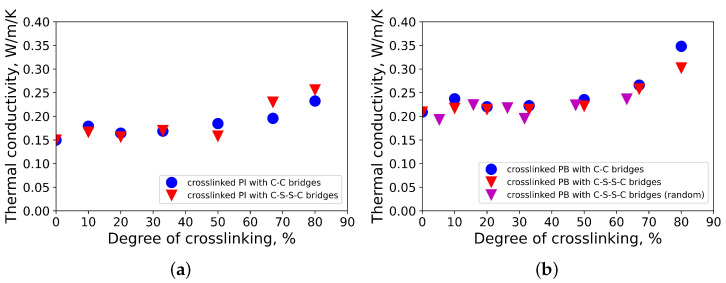
(**a**) Dependency of the thermal conductivity of crosslinked polyisoprene: red triangles and blue circles correspond to structures of vulcanized polyisoprene with C-S-S-C and C-C bridges, respectively; (**b**) dependency of thermal conductivity of crosslinked polybutadiene: red triangles and blue circles correspond to structures of vulcanized polybutadiene with C-S-S-C and C-C bridges, respectively; magenta triangles correspond to models of vulcanized polybutadiene with randomly distributed C-S-S-C bridges.

The results for the thermal conductivities of vulcanized polyisoprene and polybutadiene depending on the degree of crosslinking are presented in Figure 3. For crosslinked polyisoprene with a degree of crosslinking below 33%, a constant thermal conductivity with small fluctuations was observed (as an example, see Figure 3a). Data points from the last 0.1 ps of the last correlation time interval were taken for the calculation of the converged values of the thermal conductivities of the models of crosslinked polyisoprene.

The thermal conductivities of polyisoprene and polybutadiene increased with the increase in the degree of crosslinking, which agrees well with similar research on polyethylene [14,15], polystyrene [15], phenolic resins [16,17], and the network model [18]. Typically, polymers are inhomogeneous at high degrees of crosslinking. For example, there are hyper-crosslinked networks of polystyrene [41], polysulfones [42], polyarylates [42], poly(vinylpyridines) [43], and polyanilines [44]. However, analysis of the thermal conductivity tensors shows that in all of our simulated systems, the thermal conductivity was uniform in all directions. Moreover, the thermal conductivities of polybutadiene and polyisoprene increase slightly more at crosslinking degrees higher than 67% than at lower degrees of crosslinking. Visual changes in the simulated structures other than the number of crosslinking bridges were not observed with further crosslinking up to a crosslinking degree of 80%. The concrete type of the crosslinking bridge did not significantly influence the thermal conductivity of polyisoprene and polybutadiene. For polybutadiene, the crosslinking by C-C bridges enhanced the thermal conductivity slightly more compared to C-S-S-C bridges. These results are close to those in the literature on polybutadiene rubber (λ = 0.23 W/m/K [45]). The results for vulcanized polyisoprene are also close to those in the literature (λ = 0.14 W/m/K [45], λ ≈ 0.15 W/m/K (soft rubber) [23], and λ ≈ 0.16 W/m/K (hard rubber) [23]).

The thermal conductivity of untreated polyisoprene (λ = 0.15 W/m/K) calculated at normal conditions via the modified force field is in a good agreement with the experimental data (0.134 W/m/K [46], 0.14 W/m/K [25], and 0.145 W/m/K [47]). The result for untreated polybutadiene (λ = 0.21 W/m/K) with the modified force field is close to the experimental data (0.174 W/m/K [25], and 0.19 W/m/K [48]).

In Figure 3b, the dependence of thermal conductivity on the degree of crosslinking for vulcanized polybutadiene with randomly distributed C-S-S-C crosslink bridges is presented. The modeled vulcanized polybutadiene consists of 16 chains composed of 190 monomer units connected by randomly distributed C-S-S-C crosslinking bridges. It is obvious in Figure 3 that the type of distribution (random or uniform) of the crosslinking bridges does not significantly influence the final thermal conductivity. Due to that, only models of vulcanized polybutadiene and polyisoprene with uniformly distributed crosslinking bridges were considered in this research.

**Figure 4:**
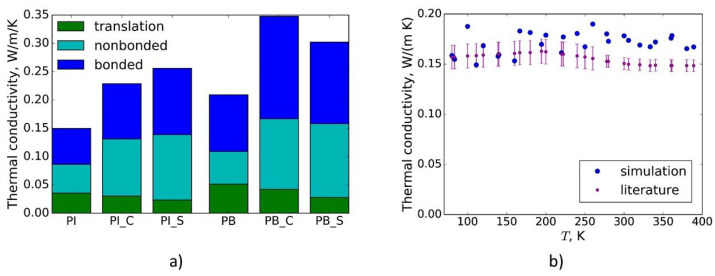
(**a**) Compositions of thermal conductivities of untreated polyisoprene (PI), vulcanized polyisoprene with C-C bridges (PI_C) with a degree of crosslinking of 80%, vulcanized polyisoprene with C-S-S-C bridges (PI_S) with degree of crosslinking 80%, untreated polybutadiene (PB), vulcanized polybutadiene with C-C bridges (PB_C) with a degree of crosslinking of 80%, and vulcanized polybutadiene with C-S-S-C bridges (PB_S) with a degree of crosslinking of 80%; (**b**) dependence of the thermal conductivity of polyisoprene crosslinked with C-S-S-C bridges (degree of crosslinking is 5.3%, which corresponds to 2.5 phr (parts per hundred rubber) of sulfur) on temperature at atmospheric pressure. For comparison, data from the literature on soft rubber from Ref. [23] are shown.

To understand the microscopic picture of heat transfer in vulcanized polybutadiene and polyisoprene, the contributions of bonded and non-bonded interactions and translational motion (convection) to the thermal conductivity were investigated. These contributions to the thermal conductivities of the modeled untreated and crosslinked polymers are shown in Figure 4a. With an increase in the degree of crosslinking, the contributions of bonded and non-bonded interactions to the thermal conductivity increase. The same was observed for crosslinked polyethylene [15].

The thermal conductivity of polyisoprene crosslinked with C-S-S-C bridges (degree of crosslinking is 5.3%, which corresponds to 2.5 phr (parts per hundred rubber) of sulfur) as a function of the temperature at atmospheric pressure is shown in Figure 4b. The results agree very well with the experimental data from Ref. [23]. Thus, MD simulations performed with the force fields used in that paper can provide the thermal conductivities of soft rubbers as a function of temperature and degree of crosslinking for macroscale simulations with FEM.

## 4. Conclusions

New modified force fields for MD simulations of polyisoprene and polybutadiene, which treat all many-body interactions, have been developed. For the first time, the thermal conductivities of polybutadiene and polyisoprene crosslinked by C-S-S-C and C-C bridges were theoretically investigated as a function of the degree of crosslinking. The dependences of the polymers’ thermal conductivities on the degree of crosslinking are in agreement with MD simulations performed for other polymeric systems. The type of crosslinking bridge studied here has no significant influence on the heat transfer in vulcanized polyisoprene and polybutadiene. The calculated values of the thermal conductivities of untreated and vulcanized systems of polybutadiene and polyisoprene agree very well with the experimental data, and the dependence of the thermal conductivity of soft rubber on temperature is close to the experimental data, too. From the analysis of the normalized heat flux autocorrelation functions of the polymers, it was found that the main mechanism of heat transfer in these polymers is through transport of low-frequency phonons, which has already been observed for other polymers. MD simulations of the modified force fields via the Green–Kubo approach can be used to obtain the thermal conductivities of crosslinked polyisoprene and polybutadiene for macroscale simulations with FEM rubbers based on these polymers.

## Data Availability

Not applicable.

## Supplementary Materials

The following are available online at https://www.mdpi.com/2073-4360/13/3/315/s1, Figure S1: (a) C-C bridge between cis-1,4-polybutadiene chains; (b) C-C bridge between cis-1,4-polyisoprene chains; (c) C-S-S-C between cis-1,4-polyisoprene chains; (d) C-S-S-C bridge between cis-1,4-polybutadiene chains, Table S1: Force field parameters for MD simulation of vulcanized polyisoprene with C-S-S-C crosslink bridges, Table S2: Force field parameters for MD simulation of vulcanized polybutadiene with C-S-S-C crosslink bridges, Table S3: Force field parameters for MD simulation of vulcanized polyisoprene with C-C crosslink bridges, Table S4: Force field parameters for MD simulation of vulcanized polybutadiene with C-C crosslink bridges.
