# Supplementary material for: Thermal Conductivities of Crosslinked Polyisoprene and Polybutadiene from Molecular Dynamics Simulations
Source: Polymers (Basel). 2021 Jan 20;13(3):315. doi: 10.3390/polym13030315 (PMC7863951; doi:10.3390/polym13030315)
Supplement: Supplementary file 1 [file polymers-13-00315-s001.pdf]

Supplementary material

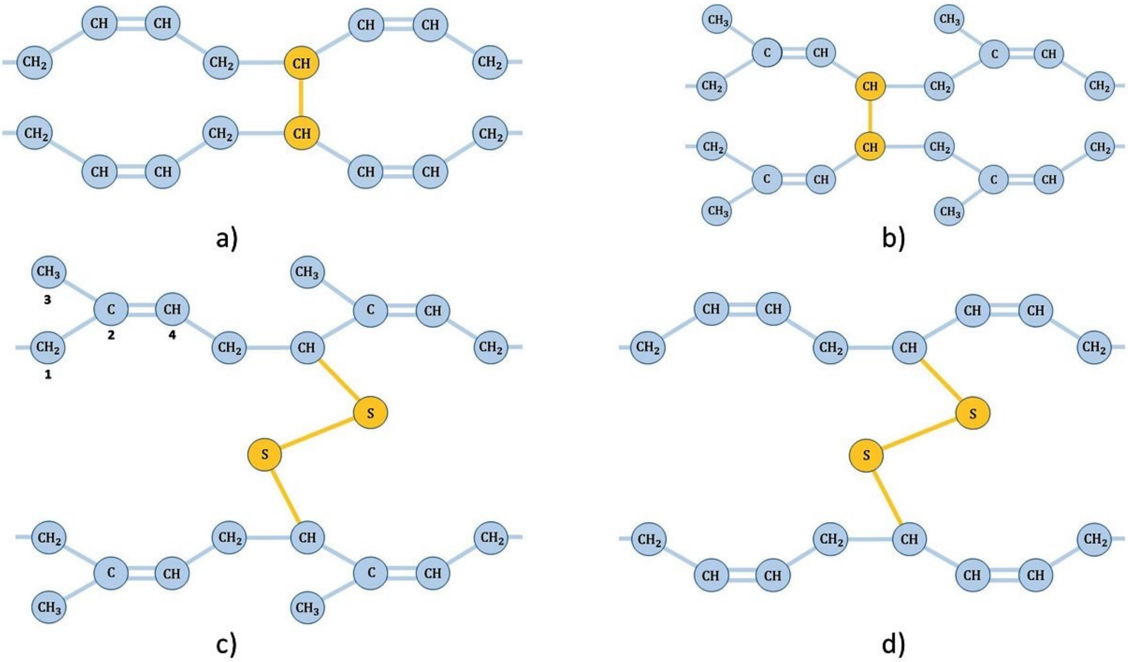

Figure S1: a) C-C bridge between *cis*-1,4-polybutadiene chains; b) C-C bridge between *cis*-1,4-polyisoprene chains; c) C-S-S-C between *cis*-1,4-polyisoprene chains; d) C-S-S-C bridge between *cis*-1,4-polybutadiene chains.

The following tables (S1-S4) show all potentials and force field parameters used for the simulations.

$E_{\mathrm{bond}} = \frac{K}{2}(r-r_0)^2$	$K[\frac{\mathrm{kcal}}{\mathrm{mol\,Å^2}}]$	$r_0[\mathrm{Å}]$	
C−CH_2	634	1.5	
C−CH_3	634	1.5	
C=CH	1060	1.34	
(=CH)−CH_2	634	1.5	
CH_2−CH_2	520	1.526	
CH_2−CH	520	1.526	
CH−C	634	1.5	
CH−S	444	1.81	
S−S	332	2.038	
$E_{\mathrm{angle}} = \frac{K}{2}(\theta-\theta_0)^2$	$K[\frac{\mathrm{kcal}}{\mathrm{mol\,rad^2}}]$	θ_0[degrees]	
CH_2−C−CH_3	140	130	
CH_2−C=CH	140	124	
CH_3−C=CH	140	124	
C=CH−CH_2	140	124	
CH−CH_2−CH_2	126	111.1	
CH_2−CH_2−C	126	111.1	
CH−CH_2−CH	126	111.1	
CH_2−CH−C	126	111.1	
CH−C−CH_3	140	130	
CH−C=CH	140	124	
CH_2−CH−S	100	114.7	
CH−S−S	136	103.7	
$E_{\mathrm{dihedral}} = \sum_{j=1}^{3}(\frac{K_j}{2}[1+(-1)^{j+1}\cos j\phi])$	$K_1[\frac{\mathrm{kcal}}{\mathrm{mol}}]$	$K_2[\frac{\mathrm{kcal}}{\mathrm{mol}}]$	$K_3[\frac{\mathrm{kcal}}{\mathrm{mol}}]$
CH_2−C=CH−CH_2	0	14	0
CH_3−C=CH−CH_2	0	14	0
C=CH−CH_2−CH_2	0.346	0.405	−0.904
CH_2−CH_2−C=CH	0.346	0.405	−0.904
CH_2−CH_2−C−CH_3	2.817	−0.169	0.543
C=CH−CH_2−CH	0.346	0.405	−0.904
CH_2−CH−C=CH	0.346	0.405	−0.904
CH_2−CH−C−CH_3	2.817	−0.169	0.543
CH−C=CH−CH_2	0	14	0
CH−S−S−CH	0	−7.414	1.705
S−CH−C−CH_3	1.711	−0.5	0.663
S−CH−C=CH	0.5	0	0
CH_2−CH−S−S	1.941	−0.836	0.935
$E_{\mathrm{improper}} = \frac{K}{2}(\chi-\chi_0)^2$	$K[\frac{\mathrm{kcal}}{\mathrm{mol\,rad^2}}]$	χ_0[degrees]	
1−4−2−3	15	180	
1−3−2−4	15	180	
3−1−2−4	15	180	
$E_{\mathrm{LJ}} = 4\epsilon[(\frac{\sigma}{r})^{12} - (\frac{\sigma}{r})^6]$	$\epsilon[\frac{\mathrm{kcal}}{\mathrm{mol}}]$	$\sigma[\mathrm{Å}]$	
CH_3 CH_3	0.145	3.96	
CH_2 CH_2	0.118	3.905	
(=CH) (=CH)	0.115	3.8	
C C	0.105	3.75	
CH CH	0.115	3.8	
S S	0.25	3.55	

Table S1: Force field parameters for MD simulation of vulcanized polyisoprene with C-S-S-C crosslink bridges.

$E_{\mathrm{bond}} = \frac{K}{2}(r - r_0)^2$	$K[\frac{\mathrm{kcal}}{\mathrm{mol\,Å^2}}]$	$r_0[\mathrm{Å}]$	
CH=CH	1060	1.34	
(=CH)−CH_2	634	1.5	
CH_2−CH_2	520	1.526	
CH_2−CH	520	1.526	
CH−S	444	1.81	
S−S	332	2.038	
$E_{\mathrm{angle}} = \frac{K}{2}(\theta - \theta_0)^2$	$K[\frac{\mathrm{kcal}}{\mathrm{mol\,rad^2}}]$	θ_0[degrees]	
CH_2−CH=CH	140	124	
CH_2−CH_2−(CH=)	126	111.1	
(=CH)−CH_2−CH	126	111.1	
CH_2−CH−(CH=)	126	111.1	
CH−CH=CH	140	124	
CH_2−CH−S	100	114.7	
CH−S−S	136	103.7	
$E_{\mathrm{dihedral}} = \sum_{j=1}^{3}(\frac{K_j}{2}[1 + (-1)^{j+1}\cos j\phi])$	$K_1[\frac{\mathrm{kcal}}{\mathrm{mol}}]$	$K_2[\frac{\mathrm{kcal}}{\mathrm{mol}}]$	$K_3[\frac{\mathrm{kcal}}{\mathrm{mol}}]$
CH_2−CH=CH−CH_2	0	14	0
CH=CH−CH_2−CH_2	0.346	0.405	−0.904
CH=CH−CH_2−CH	0.346	0.405	−0.904
CH_2−CH−CH=CH	0.346	0.405	−0.904
CH−CH=CH−CH_2	0	14	0
CH−S−S−CH	0	−7.414	1.705
S−CH−CH=CH	0.5	0	0
CH_2−CH−S−S	1.941	−0.836	0.935
$E_{\mathrm{LJ}} = 4\epsilon[(\frac{\sigma}{r})^{12} - (\frac{\sigma}{r})^6]$	$\epsilon[\frac{\mathrm{kcal}}{\mathrm{mol}}]$	$\sigma[\mathrm{Å}]$	
CH_2 CH_2	0.118	3.905	
(CH=) (CH=)	0.115	3.8	
CH CH	0.115	3.8	
S S	0.25	3.55	

Table S2: Force field parameters for MD simulation of vulcanized polybutadiene with C-S-S-C crosslink bridges.

$E_{\mathrm{bond}} = \frac{K}{2}(r - r_0)^2$	$K[\frac{\mathrm{kcal}}{\mathrm{mol\,Å^2}}]$	r_0[Å]	
C−CH$_2$	634	1.5	
C−CH$_3$	634	1.5	
C=CH	1060	1.34	
(=CH)−CH$_2$	634	1.5	
CH$_2$−CH$_2$	520	1.526	
CH$_2$−CH	520	1.526	
CH−C	634	1.5	
CH−CH	268	1.529	
$E_{\mathrm{angle}} = \frac{K}{2}(\theta - \theta_0)^2$	$K[\frac{\mathrm{kcal}}{\mathrm{mol\,rad^2}}]$	θ_0[degrees]	
CH$_2$−C−CH$_3$	140	130	
CH$_2$−C=CH	140	124	
CH$_3$−C=CH	140	124	
C=CH−CH$_2$	140	124	
C=CH−CH	140	124	
(=CH)−CH$_2$−CH$_2$	126	111.1	
CH$_2$−CH$_2$−C	126	111.1	
(=CH)−CH−CH$_2$	126	111.1	
CH−CH$_2$−(C=)	126	111.1	
CH$_2$−CH−CH	116.7	112.7	
CH−CH−(C=)	126	111.1	
$E_{\mathrm{dihedral}} = \sum_{j=1}^{3}(\frac{K_j}{2}[1 + (-1)^{j+1}\cos j\phi])$	$K_1[\frac{\mathrm{kcal}}{\mathrm{mol}}]$	$K_2[\frac{\mathrm{kcal}}{\mathrm{mol}}]$	$K_3[\frac{\mathrm{kcal}}{\mathrm{mol}}]$
CH$_2$−C=CH−CH$_2$	0	14	0
CH$_3$−C=CH−CH$_2$	0	14	0
C=CH−CH$_2$−CH$_2$	0.346	0.405	−0.904
CH$_2$−CH$_2$−C=CH	0.346	0.405	−0.904
CH$_2$−CH$_2$−C−CH$_3$	2.817	−0.169	0.543
C=CH−CH−CH$_2$	0.346	0.405	−0.904
CH−CH$_2$−C=CH	0.346	0.405	−0.904
CH−CH$_2$−C−CH$_3$	2.817	−0.169	0.543
CH$_2$−CH−CH−CH$_2$	1.3	−0.05	0.2
C=CH−CH−CH	0.346	0.405	−0.904
CH$_2$−C=CH−CH	0	14	0
CH$_3$−C=CH−CH	0	14	0
$E_{\mathrm{improper}} = \frac{K}{2}(\chi - \chi_0)^2$	$K[\frac{\mathrm{kcal}}{\mathrm{mol\,rad^2}}]$	χ_0[degrees]	
1−4−2−3	15	180	
1−3−2−4	15	180	
3−1−2−4	15	180	
$E_{\mathrm{LJ}} = 4\epsilon[(\frac{\sigma}{r})^{12} - (\frac{\sigma}{r})^6]$	$\epsilon[\frac{\mathrm{kcal}}{\mathrm{mol}}]$	σ[Å]	
CH$_3$ CH$_3$	0.145	3.96	
CH$_2$ CH$_2$	0.118	3.905	
(=CH) (=CH)	0.115	3.8	
C C	0.105	3.75	
CH CH	0.115	3.8	

Table S3: Force field parameters for MD simulation of vulcanized polyisoprene with C-C crosslink bridges.

$E_{\text{bond}} = \frac{K}{2}(r - r_0)^2$	$K[\frac{\text{kcal}}{\text{mol Å}^2}]$	r_0[Å]	
CH=CH	1060	1.34	
(=CH)−CH_2	634	1.5	
CH_2−CH_2	520	1.526	
CH_2−CH	520	1.526	
CH−CH	268	1.529	
$E_{\text{angle}} = \frac{K}{2}(\theta - \theta_0)^2$	$K[\frac{\text{kcal}}{\text{mol rad}^2}]$	θ_0[degrees]	
CH_2−CH=CH	140	124	
CH_2−CH_2−(CH=)	126	111.1	
CH−CH_2−(CH=)	126	111.1	
CH_2−CH−(CH=)	126	111.1	
CH−CH=CH	140	124	
CH_2−CH−CH	116.7	112.7	
CH−CH−(CH=)	126	111.1	
$E_{\text{dihedral}} = \sum_{j=1}^{3}(\frac{K_j}{2}[1 + (-1)^{j+1}\cos j\phi])$	$K_1[\frac{\text{kcal}}{\text{mol}}]$	$K_2[\frac{\text{kcal}}{\text{mol}}]$	$K_3[\frac{\text{kcal}}{\text{mol}}]$
CH_2−CH=CH−CH_2	0	14	0
CH=CH−CH_2−CH_2	0.346	0.405	−0.904
CH=CH−CH_2−CH	0.346	0.405	−0.904
CH_2−CH−CH=CH	0.346	0.405	−0.904
CH−CH=CH−CH_2	0	14	0
CH_2−CH−CH−CH_2	1.3	−0.05	0.2
CH−CH−CH=CH	0.346	0.405	−0.904
(=CH)−CH_2−CH−CH	1.3	−0.05	0.2
CH_2−CH−CH−(CH=)	1.3	−0.05	0.2
$E_{\text{LJ}} = 4\epsilon[(\frac{\sigma}{r})^{12} - (\frac{\sigma}{r})^6]$	$\epsilon[\frac{\text{kcal}}{\text{mol}}]$	σ[Å]	
CH_2 CH_2	0.118	3.905	
(CH=) (CH=)	0.115	3.8	
CH CH	0.115	3.8	

Table S4: Force field parameters for MD simulation of vulcanized polybutadiene with C-C crosslink bridges.
